# Discovery of Molecular Mechanisms of Traditional Chinese Medicinal Formula Si-Wu-Tang Using Gene Expression Microarray and Connectivity Map

**DOI:** 10.1371/journal.pone.0018278

**Published:** 2011-03-28

**Authors:** Zhining Wen, Zhijun Wang, Steven Wang, Ranadheer Ravula, Lun Yang, Jun Xu, Charles Wang, Zhong Zuo, Moses S. S. Chow, Leming Shi, Ying Huang

**Affiliations:** 1 National Center for Toxicological Research, U.S. Food and Drug Administration, Jefferson, Arkansas, United States of America; 2 College of Chemistry, Sichuan University, Chengdu, Sichuan, China; 3 Department of Pharmaceutical Sciences and Center for Advancement of Drug Research and Evaluation, College of Pharmacy, Western University of Health Sciences, Pomona, California, United States of America; 4 Department of Clinical Pharmacy and Center for Pharmacogenomics, School of Pharmacy, Fudan University, Shanghai, China; 5 Clinical Transcriptional Genomics Core, Medical Genetics Institute, Cedars-Sinai Medical Center, David Geffen School of Medicine at UCLA, Los Angeles, California, United States of America; 6 Functional Genomics Core, Beckman Research Institute, City of Hope Comprehensive Cancer Center, Duarte, California, United States of America; 7 School of Pharmacy, Faculty of Medicine, The Chinese University of Hong Kong, Hong Kong, China; Governmental Technical Research Centre of Finland, Finland

## Abstract

To pursue a systematic approach to discovery of mechanisms of action of traditional Chinese medicine (TCM), we used microarrays, bioinformatics and the “Connectivity Map” (CMAP) to examine TCM-induced changes in gene expression. We demonstrated that this approach can be used to elucidate new molecular targets using a model TCM herbal formula Si-Wu-Tang (SWT) which is widely used for women's health. The human breast cancer MCF-7 cells treated with 0.1 µM estradiol or 2.56 mg/ml of SWT showed dramatic gene expression changes, while no significant change was detected for ferulic acid, a known bioactive compound of SWT. Pathway analysis using differentially expressed genes related to the treatment effect identified that expression of genes in the nuclear factor erythroid 2-related factor 2 (Nrf2) cytoprotective pathway was most significantly affected by SWT, but not by estradiol or ferulic acid. The Nrf2-regulated genes *HMOX1*, *GCLC*, *GCLM*, *SLC7A11* and *NQO1* were upreguated by SWT in a dose-dependent manner, which was validated by real-time RT-PCR. Consistently, treatment with SWT and its four herbal ingredients resulted in an increased antioxidant response element (ARE)-luciferase reporter activity in MCF-7 and HEK293 cells. Furthermore, the gene expression profile of differentially expressed genes related to SWT treatment was used to compare with those of 1,309 compounds in the CMAP database. The CMAP profiles of estradiol-treated MCF-7 cells showed an excellent match with SWT treatment, consistent with SWT's widely claimed use for women's diseases and indicating a phytoestrogenic effect. The CMAP profiles of chemopreventive agents withaferin A and resveratrol also showed high similarity to the profiles of SWT. This study identified SWT as an Nrf2 activator and phytoestrogen, suggesting its use as a nontoxic chemopreventive agent, and demonstrated the feasibility of combining microarray gene expression profiling with CMAP mining to discover mechanisms of actions and to identify new health benefits of TCMs.

## Introduction

Traditional Chinese medicines (TCMs) have been used in China and other Asian countries for over 5,000 years for the prevention and treatment of a variety of diseases. In contrast to target-oriented Western medicine, TCM uses a holistic and synergistic approach to restore the balance of *Yin*-*Yang* of body energy so the body's normal function, or homeostasis, can be restored [1]. Traditional Chinese herbal medicines often consist of a combination of individual herbs to form specific formulae aimed to increase therapeutic efficacy and reduce adverse effects [2]. Theoretically, multiple active phytochemical components in the TCM formulae may simultaneously target multiple molecules/pathways and thus potentially achieve superior effect as compared to single compounds alone [3]. However, while about 100,000 herbal formulae have been recorded and there are many empiric examples of successful clinical use of TCM, relationship of the essential phytochemical components in each of the formulae to molecular targets/pathway has not been identified for most TCM due to lack of suitable methodology to tackle the complex mechanisms. Lack of molecular evidence for targets diminishes the scientific validity of the claimed usefulness of TCM, despite the availability of empiric clinical experience. Thus, new methods for molecular target/pathway identification are sorely needed to advance the modernization of TCM.

The microarray technology and associated bioinformatic data mining tools provide an opportunity to simultaneously analyze a large number of genes/targets associated with complex therapeutic effects of TCM [4]. The working principle for this genomic approach is that the phenotype of a cell, including the function and response to the environment, is ultimately determined by its gene expression profiles. Analyzing the changes of gene expression profiles after treatment by TCM in vitro or in vivo may help reveal their mechanisms of action [4], [5]. In addition, because the use of medicinal herbs may mimic or oppose the effects of concurrently used drugs, gene expression profiling using microarrays can also be used for revealing the mechanism of herb-drug interactions [6].

A few studies have used microarray-based transcriptional profiling to evaluate TCMs or their components [5], [7], [8], [9], [10]. The identified genes modulated by the TCM provided insights into molecular understanding of activity [4], [11]. However, the real challenge is to reliably detect differentially expressed genes and dissect the functional relevance of identified genes to pharmacological mechanisms from these microarray studies [12]. The improper study design and unsuitable data analysis may lead to unreliable and less accurate results derived from the microarray studies [13], [14], [15], [16]. To compare and integrate data derived from multiple different array experiments even for a single TCM component represents another technical challenge [12]. Special quality control criteria for array processing and analysis need to be used to overcome previous problems associated with microarray technologies.

Recently, the Connectivity Map (CMAP) database containing microarray expression data from cultured cell lines (e.g., human breast cancer cell line MCF-7) treated with bioactive small molecules with known mechanism of action and disease application has been described [17]. The current version of CMAP contains more than 7,000 expression profiles representing treatments from 1,309 compounds (http://www.broadinstitute.org/cMAP/) and in several studies, the CMAP database has been used for discovery of functional connections between drugs, genes, and diseases through the common gene-expression changes on the same cell lines [17], [18], [19], [20], [21]. Using CMAP, drugs affecting common molecular pathways can be identified and putative mechanism of action of unknown drugs can be explored. It may provide a useful tool for TCM when combined with microarray analysis.

**Figure 1:**
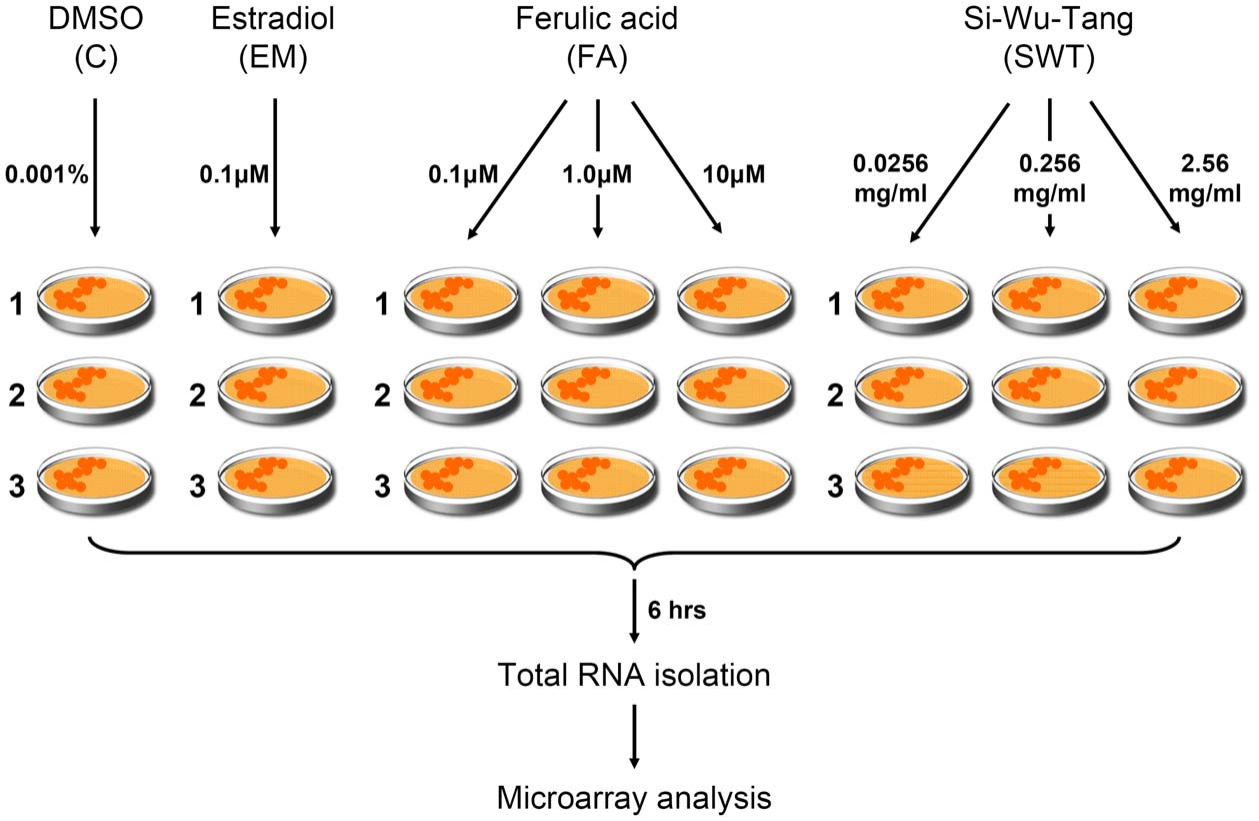
Experimental design of microarray gene expression profiling. The 24 samples were obtained from MCF-7 cells which were divided into eight treatment groups. 0.001% DMSO was used as the vehicle control (C). The cells were treated with 0.1 µM estradiol, FA at three concentrations (0.1, 1.0, and 10 µM) and SWT at three concentrations (0.0256, 0.256, and 2.56 mg/ml). For each treatment group, 3 biological replicates were included.

To demonstrate the potential application of this approach for discovery of molecular mechanisms of TCM, we studied a model TCM formula, Si-Wu-Tang (SWT, directly translated as Four Agents Decoction) [22]. SWT has been used in China and Japan for about 1,000 years for the relief of menstrual discomfort, climacteric syndrome, peri- or postmenopausal syndrome and other estrogen-related diseases [22], [23], [24], [25], [26]. It has been reported to have sedative, anti-coagulant and antibacterial activities as well as effects on vasodilatation and hematopoiesis [27]. SWT has also been shown to possess an inhibitory effect on radiation-induced bone marrow damage [27], [28]. However, the mechanism of the pharmacological action of SWT has not yet been clarified. The SWT formula is composed of four herbs, Radix *Rehmanniae praeparata*, Radix *Angelicae Sinensis*, Rhizoma *Ligustici Chuanxiong* and Radix *Paeoniae Alba* [22]. At least nine bioactive phytochemicals have been reported for SWT: paeoniflorin, paeonol, gallic acid, ferulic acid, Z-ligustilide, ligustrazine, butylphthalide, senkyunolide A and catalpol [25]. Of these compounds, ferulic acid (FA) and paeoniflorin have been recommended as the chemical markers for quality assessment of SWT [29], [30], [31], [32]. In view of wide empiric use of SWT and known chemical components already reported, in the present study, we profiled the gene expression of MCF-7 cells treated with SWT, its component FA as well as estradiol using Affymetrix microarray Human U133Plus2.0 (Figure 1). We demonstrated the feasibility of applying the combined microarray and CMAP approach in identifying molecular mechanisms of SWT.

## Results

### Hierarchical clustering analysis for quality assessment of array data and identification of treatment effects

**Figure 2:**
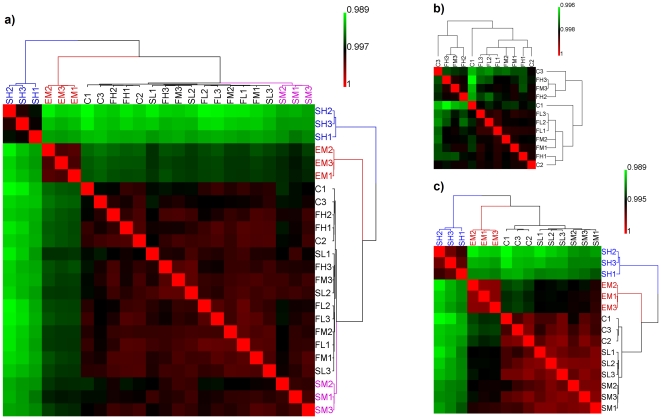
The hierarchical clustering analysis and heatmap of the correlation coefficients between gene expression profiles. (A) All 24 samples from the 8 treatment groups; (B) controls and ferulic acid treatments; and (C) controls and treatments by estradiol and SWT. There was good reproducibility between the three biological replicates in each treatment group. No clear treatment effect was observed for ferulic acid treatments. Low- and medium-concentration SWT treatments showed mild effects, while the strongest treatment effects were seen from estradiol and high-concentration SWT treatments.

Hierarchical clustering analysis was used to assess the overall quality of the microarray data (**Figure 2a**). A high correlation coefficient (colored in red in the heatmap of correlation coefficients) means that the gene expression profiles from two microarrays are very similar. The three replicates in each treatment group showed high pair-wise correlation in terms of log2 gene expression. In addition, samples treated with estradiol and high concentration of SWT showed dramatically different expression profiles compared to that of the control group. The visual observation of the clustering results indicates satisfactory reproducibility of microarray experiments for the biological replicates in each treatment group and significant treatment effects of estradiol and SWT, warranting further analyses and interpretation of their treatment effects.

Three replicates of MCF-7 control and MCF-7 treated with ferulic acid at low, medium and high concentrations were used for hierarchical clustering analysis in **Figure 2b**. For every dose-treatment group, the three replicates in the same group did not cluster together, indicating that there was no clear treatment effect even at the high concentration of ferulic acid. It seems that the treatment effects of ferulic acid on MCF-7 are minimal at the doses tested in this study.

In inspecting the treatment effects of SWT at low, medium and high concentrations along with the three replicates of estradiol treatment, hierarchical clustering analysis (**Figure 2c**) clearly demonstrated the high reproducibility of the replicates in each treatment group and strong treatment effects for estradiol and SWT at high concentration. The figure also shows clustering of the three replicates in the medium or low concentration of SWT treatment, but the degree of the treatment effect was much smaller than that for high-concentration SWT treatment. The expression profiles for the three replicates in the estradiol treatment group also clustered tightly together and were dramatically different from those of the control group samples. The expression profiles of the estradiol group were also dramatically different from those of the high-concentration SWT treatment group.

### Identification of differentially expressed genes

Because there are 54,675 probe sets on the Affymetrix Human U133Plus 2.0 microarray, 2,734 probe sets would be expected by chance to show a *p*<0.05. By comparing the number of probe sets with a *p*<0.05 for each treatment group (**Table 1**) with that expected by chance (2,734), we can roughly assess the degree of the treatment effect. By applying the same cutoffs of t-test *p* value <0.05 and fold change >1.5, the differentially expressed genes were selected separately from comparing each treatment group with the control group. The number of differentially expressed genes varied dramatically depending on the treatment group. The high-concentration SWT and estradiol treatments resulted in the highest numbers of genes differentially expressed, whereas the treatment effect of ferulic acid appeared to be minor for all concentrations, and there was no clear dose-response relationship. On the other hand, the numbers of genes differentially expressed as a result of SWT treatment showed a clear dose-response trend, with an increase from low dose (7 genes, corresponding to 10 probe sets) to medium dose (71 genes, corresponding to 90 probe sets) and a big jump from medium dose to high dose (1,911 genes, corresponding to 2,979 probe sets). The treatment with estradiol (0.1 µM) changed the expression of 830 unique genes (corresponding to 1,292 probe sets). However, only 337 genes were commonly regulated by both estradiol treatment and the high-concentration SWT treatment, indicating similarities and differences in the mechanisms of action between the two agents. Consistently, by controlling the false discovery rate (FDR) at the level of 0.05, only the high-concentration SWT and estradiol treatments resulted in genes differentially expressed (**Table 1**).

**Table 1:** Treatment information and the number of differentially expressed genes of each treatment group.

Treatment	Concentration	Hybridization name	No. of probe sets (*p*<0.05, FC>1.5)	No. of genes (*p*<0.05, FC>1.5)	No. of probe sets(*p*<0.05)*	No. of probe sets (*FDR*<0.05)
Control	-	C1, C2, C3	-	-	-	-
Estradiol	0.1 µM	EM1, EM2, EM3	1,292	830	11,595	3,598
Ferulic acid	0.1 µM	FL1, FL2, FL3	9	8	2,965	0
	1 µM	FM1, FM2, FM3	6	4	3,332	0
	10 µM	FH1, FH2, FH3	3	3	2,270	0
SWT	0.0256 mg/mL	SL1, SL2, SL3	10	7	3,578	0
	0.256 mg/mL	SM1, SM2, SM3	90	71	5,409	0
	2.56 mg/mL	SH1, SH2, SH3	2,979	1911	13,296	6,673

*For the U133Plus2 microarrays with 54,675 probe sets, 2,734 (54,675×0.05) probe sets are expected by chance to have a *p* value <0.05.

FDR: false discovery rate.

### Canonical pathway analysis by IPA software: the Nrf2-mediated oxidative stress response pathway is most significantly impacted by high-concentration SWT treatment

**Figure 3:**
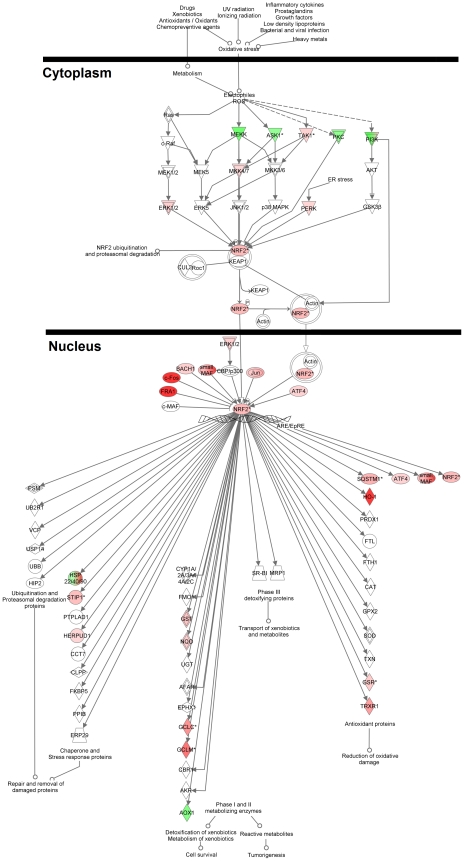
The Nrf2-mediated oxidative stress response pathway in IPA database. The red color and green color indicate the up- and down-regulated genes after treatment with high-concentration of SWT in this pathway, respectively.

The pathway names, Fisher's exact test *p* values, and the ratios of impacted genes for the top 10 IPA pathways most significantly enriched with genes differentially expressed from SWT and estradiol treatments are listed in **Table 2**. For the low-concentration SWT treatment, none of the IPA pathways was significantly enriched, mainly because of the very small number of differentially expressed genes (10 probe sets, corresponding to 7 genes). For medium-concentration SWT treatment, several IPA pathways related to the metabolism of xenobiotics by CYP450, C21-steroid hormone metabolism, cell cycle, and molecular mechanisms of cancer were highly enriched with differentially expressed genes. Genes differentially expressed from high-concentration SWT treatment group were most significantly enriched in several cancer signaling pathways. In particular, the Nrf2-mediated oxidative stress response pathway, which has recently been found to play an important role in cancer prevention, was the most significantly impacted pathway (*p* = 4.55×10^−9^). About 23% (42/183) of the genes in the Nrf2 pathway were either up- or down-regulated by high-concentration SWT treatment (**Figure 3**).

**Table 2:** Top IPA pathways enriched with differentially expressed genes and their corresponding Fisher's exact test *p* values.

Treatment	IPA pathway name	*p*-value	Total number of genes in the IPA pathway	Ratio*
SWT at high concentration	Nrf2-mediated Oxidative Stress Response	4.55e-09	183	0.230
	p53 Signaling	2.37e-07	92	0.283
	Molecular Mechanisms of Cancer	6.17e-07	372	0.164
	Glucocorticoid Receptor Signaling	3.68e-06	280	0.168
	CD40 Signaling	2.92e-05	67	0.254
	Pancreatic Adenocarcinoma Signaling	3.26e-05	116	0.207
	EGF Signaling	3.26e-05	49	0.286
	B Cell Receptor Signaling	7.57e-05	154	0.188
	TGF-β Signaling	8.81e-05	83	0.229
	HGF Signaling	1.22e-04	103	0.214
SWT at medium concentration	Cell Cycle: G1/S Checkpoint Regulation	4.28e-05	59	0.068
	Role of CHK Proteins in Cell Cycle Checkpoint Control	2.27e-04	35	0.086
	Aryl Hydrocarbon Receptor Signaling	1.18e-03	154	0.026
	Molecular Mechanisms of Cancer	1.21e-03	372	0.016
	C21-Steroid Hormone Metabolism	2.11e-03	71	0.028
	Metabolism of Xenobiotics by Cytochrome P450	3.47e-03	209	0.014
	TR/RXR Activation	3.71e-03	97	0.031
	p53 Signaling	4.22e-03	92	0.033
	Cell Cycle Regulation by BTG Family Proteins	7.06e-03	36	0.056
	ATM Signaling	1.46e-02	53	0.038
SWT at low concentration	None of the pathways was significantly enriched with differentially expressed genes.			
Estradiol	Role of BRCA1 in DNA Damage Response	5.10e-09	61	0.246
	Cell Cycle: G1/S Checkpoint Regulation	2.69e-07	59	0.220
	Role of CHK Proteins in Cell Cycle Checkpoint Control	6.22e-06	35	0.257
	Aryl Hydrocarbon Receptor Signaling	7.09e-06	154	0.117
	Hereditary Breast Cancer Signaling	1.85e-05	129	0.124
	Molecular Mechanisms of Cancer	2.80e-05	372	0.083
	Pyrimidine Metabolism	5.74e-05	231	0.078
	Glycosphingolipid Biosynthesis - Globoseries	3.86e-04	46	0.130
	p53 Signaling	3.87e-04	92	0.130
	Pancreatic Adenocarcinoma Signaling	1.21e-03	116	0.103

*The ratio is calculated by dividing the number of differentially expressed genes found in the pathway by the total number of genes involved in the pathway.

We also used the expression profile of estradiol treatment as the query to search the IPA pathway database. The top 10 hits (**Table 2**) include pathways related to cell cycle, molecular mechanisms of cancer, and p53 signaling. These pathways are well-known to be associated with the biological functions of estradiol, validating the quality of microarray gene expression data in this study and the utility of the IPA pathway analysis approach. Notably, although the Nrf2-mediated oxidative stress response pathway was significantly impacted by high-concentration SWT treatment, it was not affected by estradiol treatment at the condition tested in this study.

### Pathway analysis in KEGG database

None of the KEGG pathways was significantly enriched with the differentially expressed genes from low-concentration SWT treatment group and only 8 KEGG pathways were significantly enriched from medium-concentration SWT treatment. Instead, there were 25 and 19 KEGG pathways significantly enriched with differentially expressed genes from high-concentration SWT treatment and estradiol treatment, respectively. The top 10 significantly impacted KEGG pathways were listed in **Table 3**. Comparing the KEGG pathways listed in **Table 3** with the IPA pathways listed in **Table 2**, we can see that pathways related to signaling of cancer, cell cycle and metabolism were significantly impacted by estradiol treatment and by SWT treatments at medium- and high-concentrations, highlighting the commonalities between the pharmacological effects of estradiol and SWT.

**Table 3:** Top KEGG pathways enriched with differentially expressed genes and their corresponding Fisher's exact test *p* values.

Treatment	KEGG pathway name (Entry ID)	*p*-value
SWT at high concentration	MAPK signaling pathway (hsa04010)	4.81e-05
	TGF-beta signaling pathway (hsa04350)	7.76e-05
	Colorectal cancer (hsa05210)	9.76e-05
	Acute myeloid leukemia (hsa05221)	1.26e-04
	p53 signaling pathway (hsa04115)	8.88e-04
	Apoptosis (hsa04210)	9.54e-04
	Axon guidance (hsa04360)	1.28e-03
	Bladder cancer (hsa05219)	1.52e-03
	Glutamate metabolism (hsa00251)	2.60e-03
	Chronic myeloid leukemia (hsa05220)	2.73e-03
SWT at medium concentration	p53 signaling pathway (hsa04115)	4.36e-04
	Bladder cancer (hsa05219)	1.42e-03
	Prion disease (hsa05060)	2.49e-03
	Cell cycle (hsa04110)	3.12e-03
	Metabolism of xenobiotics by cytochrome P450 (hsa00980)	6.13e-03
	Small cell lung cancer (hsa05222)	1.12e-02
	Tetrachloroethene degradation (hsa00625)	3.76e-02
	Acute myeloid leukemia (hsa05221)	3.79e-02
SWT at low concentration	None of the pathways was significantly enriched with differentially expressed genes.	
Estradiol	Cell cycle (hsa04110)	1.00e-08
	Purine metabolism (hsa00230)	5.69e-04
	Porphyrin and chlorophyll metabolism (hsa00860)	3.18e-03
	Glycosphingolipid biosynthesis - globoseries (hsa00603)	3.70e-03
	p53 signaling pathway (hsa04115)	5.18e-03
	Pentose and glucuronate interconversions (hsa00040)	6.21e-03
	One carbon pool by folate (hsa00670)	6.24e-03
	Pyrimidine metabolism (hsa00240)	9.69e-03
	Thyroid cancer (hsa05216)	1.19e-02
	Bladder cancer (hsa05219)	1.49e-02

### Identification of dose-responsive genes and pathways

**Figure 4:**
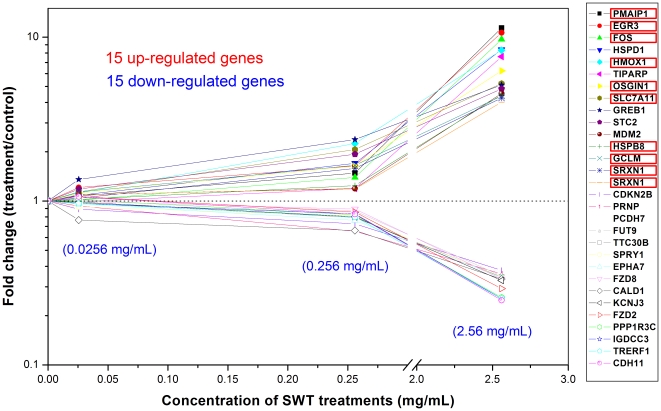
Dose-responsive genes with the largest fold changes. Among 15 dose-responsive up-regulated genes (probe sets) with the largest fold changes, ten are related to Nrf2 (highlighted in red box) according to PubMed literature search.

Since genes or pathways that show dose-dependent changes are most likely reflecting valid pharmacological action from a drug, we therefore created two lists of differentially expressed genes (probe sets, *p*<0.05) from SWT treatments at medium and high concentrations in comparison to the controls and identified common genes by overlapping the two lists. From this list of common genes, we can determine dose-responsive genes for which the expression level increased or decreased and mapped these to unique gene symbols in the IPA software. In total, 1,240 unique genes were identified as dose-responsive genes that are associated with the pathways in the IPA software. The intensity of these genes obviously increased or decreased when the concentration of SWT treatments increased from 0.0256 mg/mL (SL) to 2.56 mg/mL (SH). **Table 4** lists the names, Fisher's exact test *p* values, and the ratios of affected genes for the top 10 IPA pathways most significantly enriched with the dose-responsive genes to SWT. The Nrf2 pathway is again on the top of list. Among all the 1,240 genes showing dose-responsive changes, 24 upregulated and 7 downregulated genes could be assigned to the Nrf2 pathway. **Figure 4** showed that 10 out of the15 most upregulated genes (probe sets) are regulated by the Nrf2 pathway according to PubMed literature search. Because of recently reported cancer prevention role of the Nrf2 pathway, our data suggest that SWT may have an Nrf2-inducing activity and may possess cancer chemopreventive effects.

**Table 4:** Top IPA pathways enriched with genes showing dose-responsive changes after SWT treatment and corresponding Fisher's exact test *p* values.

Ingenuity canonical pathways	p value	Total number of genes in IPA pathway	Ratio[Table-fn nt104]
Nrf2-mediated Oxidative Stress Response	4.9E-05	183	0.169
Chronic Myeloid Leukemia Signaling	0.0001	105	0.190
PPAR Signaling	0.0001	98	0.194
Glutamate Metabolism	0.0002	78	0.141
Cell Cycle: G1/S Checkpoint Regulation	0.0002	59	0.237
Small Cell Lung Cancer Signaling	0.0003	89	0.180
Protein Ubiquitination Pathway	0.0003	201	0.149
Molecular Mechanisms of Cancer	0.0004	372	0.126
mTOR Signaling	0.0005	156	0.154
PI3K/AKT Signaling	0.0007	137	0.153

*The ratio is calculated by dividing the number of differentially expressed genes found in the pathway by the total number of genes involved in the pathway.

### Mapping gene expression profiles to the CMAP reference database

Since the same cell line (MCF-7) and same treatment period (6 h) were used to generate the above dataset as the CMAP dataset, comparison analysis can be done between the two datasets. Estradiol is the common drug used in both datasets. Thus, to further evaluate the quality and usability of the gene expression data generated in this study, we first used the gene expression profile from estradiol treatment as a query to search the CMAP database. If our data are in good quality and the CMAP approach works, we should expect the gene expression profiles of MCF-7 cells treated with estradiol in CMAP to show up on the list of top hits. Indeed, when the top 200 (100 up-regulated and 100 down-regulated) differentially expressed genes were used as the query, the CMAP profile of estradiol treated MCF-7 cells surfaced on the top 10 list (**Table 5**). In addition, the gene expression profiles resulting from treatments of three compounds, including butyl hydroxybenzoate, alpha-estradiol and genistein, were also on the top of the hit list. These compounds have been known to share the same molecular mechanisms of action with estradiol, mainly by regulating the hormonal signal transduction systems. Interestingly, the profile from treatment with fulvestrant, also known as ICI 182,780, turned out to be most contradictory to that from estradiol in our study, with a mean score of -0.806. This finding is consistent with the mechanistic role of fulvestrant, a pure estrogen receptor antagonist with no agonist effect for treating hormone receptor-positive metastatic breast cancer in postmenopausal women. These results from estradiol treatment indicated the value of the CMAP reference database and enhanced our confidence in the reliability of the microarray data from our study.

**Table 5:** Top CMAP hits correlated with SWT or estradiol treatment.

Treatment*	CMap chemical name and cell line	Mean score	*p*-value
SWT at high concentration	Phenoxybenzamine - MCF7	0.964	<0.00001
	Withaferin A - MCF7	0.885	<0.00001
	Securinine - MCF7	0.781	0.00002
	15-delta prostaglandin J2 - MCF7	0.697	<0.00001
	Thioridazine - MCF7	0.436	<0.00001
	Resveratrol - MCF7	0.432	<0.00001
	Estradiol - MCF7	0.345	<0.00001
	Tanespimycin - MCF7	0.188	<0.00001
	0317956-0000 - MCF7	−0.58	<0.00001
	Fulvestrant - MCF7	−0.698	<0.00001
SWT at medium concentration	Estradiol - MCF7	0.504	<0.00001
	Genistein - MCF7	0.452	<0.00001
	Valproic acid - MCF7	−0.367	0.00008
	Trichostatin A - HL60	−0.392	<0.00001
	LY-294002 – HL60	−0.428	<0.00001
	LY-294002 – MCF7	−0.445	0.00004
	Sirolimus - MCF7	−0.493	<0.00001
	Trichostatin A - MCF7	−0.658	<0.00001
	Vorinostat - MCF7	−0.608	0.00002
	Fulvestrant - MCF7	−0.765	<0.00001
SWT at low concentration	There were no down-regulated genes and CMap search could not be performed.		
Estradiol	Butyl hydroxybenzoate - MCF7	0.826	<0.00001
	Estradiol - MCF7	0.667	<0.00001
	Genistein - MCF7	0.632	<0.00001
	Alpha-estradiol - MCF7	0.544	<0.00001
	Trichostatin A - MCF7	−0.427	<0.00001
	0317956-0000 - MCF7	−0.434	<0.00001
	Vorinostat - MCF7	−0.457	<0.00001
	Phenoxybenzamine - MCF7	−0.496	<0.00001
	Pyrvinium - MCF7	−0.561	<0.00001
	Fulvestrant - MCF7	−0.806	<0.00001

*For high-concentration SWT and estradiol treatments, the query to CMAP search included 100 up-regulated and 100 down-regulated genes. For medium-concentration SWT treatment, all the 53 differentially expressed genes (3 down-regulated and 50 up-regulated) were used as the query for CMAP search.

We also used the gene expression profiles of 100 up-regulated and 100 down-regulated genes from high-concentration SWT treatment and all the 53 differentially expressed genes from medium-concentration SWT treatment to query the CMAP database. For the low-concentration SWT treatment, CMAP search could not be performed because there were no down-regulated genes. The CMAP gene expression profile of MCF-7 treated with estradiol was the only one showing significant match (permutation *p*<0.00001) for both high- and medium-concentration SWT, indicating that SWT has an effect on MCF-7 cells similar to that of estradiol. This finding appeared to be consistent with SWT's widely claimed use as a TCM for women's diseases. The CMAP profiles of several other compounds (including phenoxybenzamine, withaferin A, 15-delta prostaglandin J2 and resveratrol) also showed high similarity to that of high-concentration SWT treatment. The pharmacological effects of these compounds are not similar to that of estradiol, suggesting that SWT has additional effects not seen with estradiol.

### Validation of the microarray gene expression data by real-time RT-PCR

The differential expression of five genes in the Nrf2 pathway in response to SWT was validated by quantitative real-time RT-PCR on samples obtained from MCF-7 cells treated in a separate experiment. The selected genes are *HMOX1* (Heme oxygenase 1, HO-1), *GCLC* (glutamate-cysteine ligase, catalytic subunit), *GCLM* (glutamate-cysteine ligase, modifier subunit), *NQO1* (NAD(P)H:quinine oxidoreductase 1) and *SLC7A11* [solute carrier family 7, (cationic amino acid transporter, y+ system) member 11]. The fold changes of expression determined by RT-PCR for these genes were concordant with those obtained by microarrays (**Table 6**). The magnitude of the fold change from the RT-PCR assay was greater than that from the microarrays.

**Table 6:** The gene expression fold changes of RT-PCR in comparison with the microarrays.

	SL		SM		SH	
Gene	Microarray	RT-PCR	Microarray	RT-PCR	Microarray	RT-PCR
*SLC7A11*	1.14±0.09	0.80±0.03	2.07±0.17**	1.33±0.16*	5.21±0.43**	8.38±0.13**
*HMOX1*	1.13±0.14	1.63±0.07	2.25±0.11**	5.73±0.55*	8.35±0.12**	45.99±0.53**
*GCLC*	1.01±0.08	3.58±0.12	1.42±0.02**	4.01±0.14*	3.12±0.06**	17.27±0.03**
*GCLM*	1.14±0.09*	1.06±0.12	1.65±0.07*	1.31±0.04*	4.38±0.33**	6.32±0.11**
*NQO1*	0.98±0.01	1.20	1.08±0.03	1.39	1.01±0.04	2.28

*P<0.05.

**P<0.01.

The standard deviation (SD) is not applicable for *NQO1* gene, for which the PCR was performed for one time.

### Effects of SWT on activation of Nrf2/ARE by dual luciferase reporter gene assay

**Figure 5:**
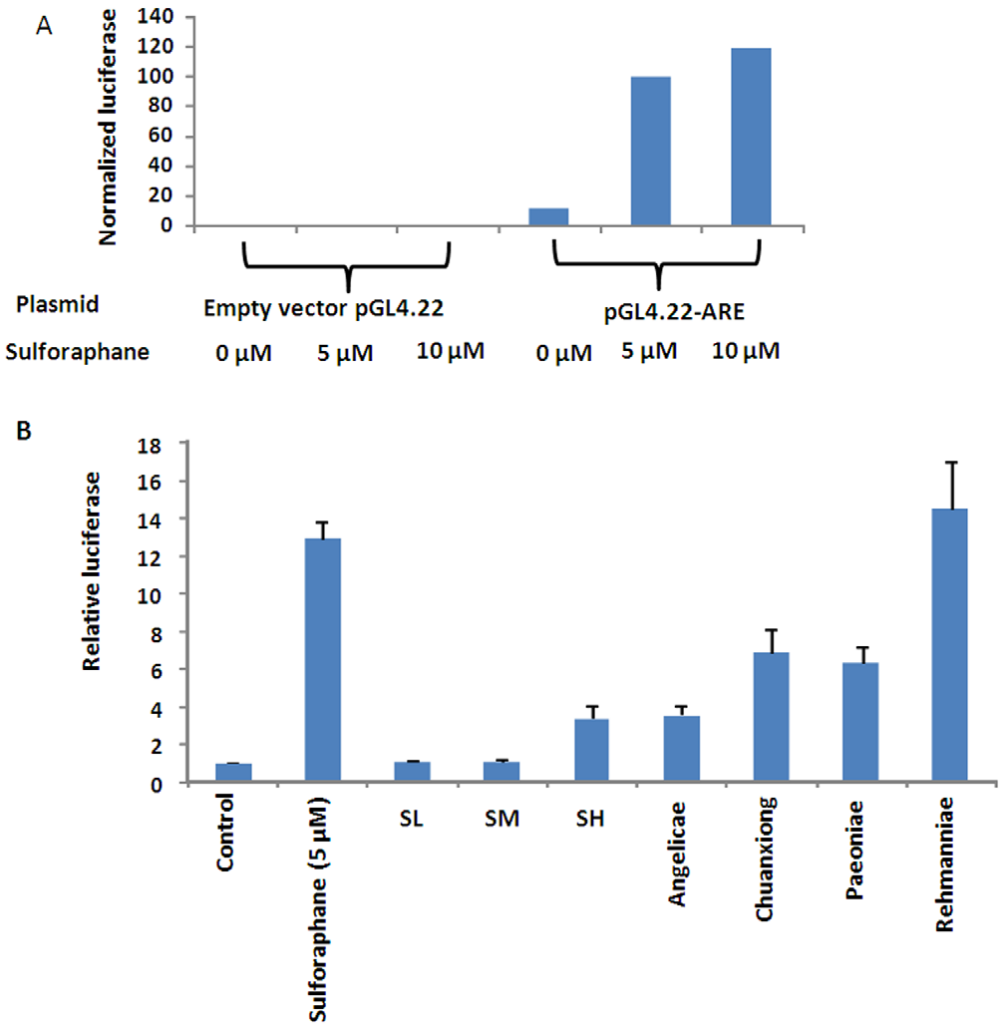
Luciferase assay results. (A) Luciferase assay established using HEK-293 cells co-transfected with a plasmid containing an ARE-luciferase reporter gene (pGL4.22-ARE) or empty vector (pGL4.22) and a plasmid encoding renillar luciferase (pGL4.74). The transfected cells were treated with sulforaphane for 24 hr prior to measurement of firefly and renillar luciferase activities using the dual luciferase reporter gene assay. (B) SWT (three doses SL, SM and SH in 0.0256, 0.256 and 2.56 mg/ml) and four herbal components of SWT (2.56 mg/ml) activated the Nrf2/ARE signaling pathway in MCF-7 cells.

In order to measure the activation of the Nrf2 pathway, a 39-bp ARE-containing sequence from the promoter region of the human *NQO1* gene was inserted into the cloning site of the luciferase plasmid pGL4.22 and then transiently transfected into MCF-7 cells. This assay was firstly established using HEK293 cells with sulforaphane as a positive control (**Figure 5A**). The transfected cells were then treated with SWT (SL, SM and SH) and the four individual herbal ingredients. A dual luciferase reporter gene assay was used in which a renilla luciferase gene was used as an internal control for normalization of the transfection efficiency and for toxicity induced during drug exposure. The high concentration of SWT (SH) showed 3.4±0.68-fold increase of the luciferase activity, while the individual herbs increased the luciferase activity to a higher degree (**Figure 5B**). Rehmanniae was found to be strongest activator for Nrf2/ARE transcription. Similar results have been obtained in another cell line HEK293 cells (data not shown). These results suggest that SWT and its ingredients may upregulate the expression of the Nrf2 target genes by activating the ARE on the promoters of these genes.

## Discussion

The use of TCM is widespread in China and many Asian countries, and is also rapidly growing in Western countries [3]. Despite its long history of use, many questions remain to be answered due to lack of mechanistic understanding. In this study, we demonstrated, for the first time, a combined microarray gene expression and CMAP data mining approach to discover the mechanisms of action and to identify new therapeutic uses for TCM. We used a popular TCM formula SWT as a model to demonstrate the potential application of this approach.

The array data for 24 samples were firstly analyzed by hierarchical clustering analysis for a quality assessment of the array data and for a comparison of the treatment effects. The three biological replicates in each treatment group showed a high reproducibility in the microarray experiments. The clustering results and the numbers of differentially expressed genes in each treatment group also revealed that the gene expression profile of MCF-7 cells was strongly changed by the treatment with estradiol and high-concentration of SWT, but not by FA and low-concentration SWT. The pathway analysis identified the Nrf2-mediated oxidative stress response as the pathway most significantly changed among differentially expressed genes showing dose-dependent response to SWT treatment. This new finding suggests that SWT could be cancer preventive. The real-time PCR data showed a similar but higher degree of gene upregulation of select genes in the Nrf2 pathway. When the gene expression profiles of MCF-7 cells resulting from SWT treatment were used to compare with those from 1,309 compounds in the CMAP database, the CMAP gene expression profile of estradiol-treated MCF-7 cells turned out to be the best match, consistent with SWT's widely claimed use for women's diseases and suggesting a potential phytoestrogenic effect. The CMAP profiles of several compounds with chemopreventive activity, i.e., withaferin A and resveratrol, also showed high similarity to the profiles of SWT.

One interesting finding is that the expression of genes involved in the Nrf2 signaling pathway was strongly impacted by SWT, but not by estradiol or ferulic acid treatment. The Nrf2 (nuclear factor erythroid 2 -related factor 2), a basic zip (bZIP) transcription factor, has been known as a key molecular target for chemopreventive agents, in particular, natural products and phytochemicals with activities in chemoprevention (for reviews, see [33] and [34]). Nrf2 plays a central role in the regulation of basal and/or inducible expression of phase II genes by binding to the antioxidant response elements (AREs) in their promoters [35]. Nrf2 is normally sequestered in the cytoplasm by Kelch-like ECH-associated protein 1 (Keap1). When activated upon exposure to inducers, it dissociates from Keap1, translocates to the nucleus, complexes with other nuclear factors, and binds to the ARE of genes. Chemopreventive Nrf2 inducers affect the interaction between Keap1 and Nrf2 through several mechanisms such as redox-sensitive modifications on cysteine residues of Keap1 or other cellular sensors and phosphorylation of Nrf2 [36]. Consistent with our results, the extract of *Angelicae Sinensis* (Dang Gui), one of the four herbal ingredients of SWT, has been shown to induce the expression of the detoxification enzyme NAD(P)H:quinine oxidoreductase 1 (NQO1), which is regulated by the Nrf2 pathway [37]. One of the active compounds, Z-ligustilide, was found with strong NQO1 inducing property by alkylating Keap1 [37]. We have also tested the effects of Z-liguistilide treatment on MCF-7 cells on Nrf2 gene expression and obtained the similar inducing effects (unpublished results).

The Nrf2-inducing activity of SWT represents an intriguing and interesting finding. SWT and its components, by affecting multiple Nrf2 target genes, could impact multiple components of the carcinogenic process. Many of the downstream target genes of Nrf2 are important in maintaining the cellular antioxidative response and amelioration of oxidative stress [38]. For example, the glutamate-cysteine ligase and SLC7A11 are essential in regulating the synthesis of glutathione, a very powerful endogenous antioxidant [39]. The NAD(P)H quinone oxidoreductase 1 (NQO1) catalyzes the reduction and detoxification of highly reactive quinones that can cause oxidative stress [40]. HO-1 (HMOX1) has been shown to protect from a variety of pathologies, including sepsis, hypertension, atherosclerosis, acute lung injury, kidney injury, and pain [41]. In addition, the effect of SWT on cytoprotective and detoxification pathways may explain other clinical effects of SWT. For example, its bone marrow protective effect on irradiated mice [27], [28], [42] may be attributed to the Nrf2-induding effects which may increase the radioresistance of bone marrow stem cells. It is also noted that many of Nrf2-regulated genes showed dose-dependent upregulation by SWT, but not by estrogen or ferulic acid. This suggests that the Nrf-2 activating effects of SWT may be separated from its phytoestrogenic effect. Further studies are needed to clarify the effect from different compounds compared to the whole SWT formula.

The identified differentially expressed genes in each treatment group were used in the comparison with the CMAP reference database [17]. We can directly compare the expression profiles of estradiol in our dataset and with those in the CMAP dataset due to the same cell line (MCF-7) and treatment time (6 hours). The gene expression profiles of SWT showed an excellent match to the CMAP gene expression profile of estradiol-treated MCF-7 cells (permutation *p*<0.00001). This finding is consistent with SWT's widely claimed use as an effective traditional Chinese medicine for women's diseases with a possible phytoestrogenic effect. The profile of SWT also shows similarity to the CMAP profiles of withaferin A, 15-delta prostaglandin J2 and resveratrol, which have been reported to possess chemopreventive effects [43], [44]. Results of KEGG pathway analysis found SWT and estradiol shared certain commonly regulated pathways of “cell cycle regulation”, “p53 signaling” and “molecular mechanisms of cancer”, further supporting SWT's phytoestrogenic effects. However, the gene expression profiles for SWT and estradiol also showed a significant difference. One of the most notable differences is that the ability to alter gene expression in the Nrf2 pathway only exists for SWT, but not for estradiol. The role of estrogens in the initiation and progression of breast cancer has been well known [45]. However, there is a large body of evidence that the consumption of phytoestrogens derived from plants and TCM can decrease the risk of cancer although they display estrogen-like activity [46]. These results support a notion that SWT may not have the cancer-causing effects of estradiol, but only have the beneficial cell protective activity.

Based on our previous work [25], the content of FA of the SWT extract was found to be 0.076%, corresponding to 0.0194, 0.194, and 1.94 µg/ml of FA, which were the concentrations tested in the present study. Such dosage selection allows a comparison of the activity of single compound (FA) with that of the TCM formulation (SWT). However, our data showed that FA in all the concentration tested only had subtle effects on global transcription in MCF-7 cells, although FA has been suggested as the chemical fingerprint for quality control of SWT products [29], [30], [31]. In view of lack of activity on gene expression, FA should not be used as a biological marker for activity of SWT.

The lack of expected gene expression change by FA may partially be explained by the statistical limitations of the approach: the sample size (n = 3 for each treatment group) and the fact that multiple tests of significance being done simultaneously may contribute to false positive/negative findings. Although the “multiple testing” corrections by False Discovery Rate (FDR) analysis were described in the text and Methods, such criteria may be too stringent to identify differentially expressed genes from treatment groups of FA and low concentration of SWT (Table 1). The biological limitations for this study, as is the case for almost all transcript profiling studies, is that there remains uncertainty about the relationship between mRNA and protein expression, and the relationship of both to function. Nevertheless, as indicated in Figure 5, a functional assay of Nrf2 pathway strongly supports the results obtained from microarray analysis.

In conclusion, gene expression profiles obtained by using microarrays combined with bioinformatic mining of the CMAP reference database can potentially shed light on the new molecular mechanism of SWT. If SWT can be shown to have cancer preventive potential, it has a great economic advantage because of its low cost and low toxicity, which will have a profound impact on human health. Further work is needed to determine the in vivo relevance of the in vitro findings obtained from the present study. This new approach proved to be powerful in an understanding of mechanisms of actions for TCM as exemplified by our study with SWT. In addition, the gene expression changes identified in this study could be used as biomarkers which can be used for assessing the intact quality of SWT. The genomic approach can be integrated with traditional chromatography-based fingerprinting method and metabolomics to obtain a more complete understanding of TCMs.

## Materials and Methods

### Compounds

Ferulic acid, 17 β-estradiol and DMSO were purchased from Sigma-Aldrich (St. Louis, MO, USA).

### Preparation of SWT extracts

The SWT products were obtained from the School of Pharmacy, Chinese University of Hong Kong. These products were manufactured under GMP condition at the Hong Kong Institute of Biotechnology (Hong Kong, China) according to the protocol described in Chinese Pharmacopoeia 2005 [47] with modifications. Crude water extracts were prepared from powdered SWT. Fresh extracts were prepared right before the experiment. Three concentrations of SWT extract were prepared, 2.56 mg/ml, 0.256 mg/ml and 0.0256 mg/ml for high (SH), medium (SM), and low (SL) concentrations, respectively. The SH was prepared by dissolving 2.56 mg SWT powder into 1 ml of PBS buffer, followed by sonication for 30 min. The SM and SL were prepared by dilute the SH 10 and 100 times using PBS, respectively. As determined previously [25], the SL, SM and SH solutions contain 0.0194, 0.194, and 1.94 mg/ml of ferulic acid, respectively.

### Cell lines and cell culture

The MCF-7 cells were purchased from American Type Culture Collection (ATCC, Manassas, VA, USA), cultured in Dulbecco's modified Eagle's medium (DMEM) supplemented with 10% fetal bovine serum (FBS), 1% non-essential amino acids, 100 unit/mL penicillin, 100 µg/mL streptomycin, 1 mM sodium pyruvate, and 2 mM L-glutamine in an atmosphere of 5% CO_2_ at 37°C. The cells were seeded in 6-well plates at a density of 1×10^5^ cells/ml. After incubating for 24 hours and at least 4 days before treatment, the medium was then replaced by hormone free medium which contains phenol-red free DMEM medium supplemented with 5% charcoal-dextrin stripped FBS (CD-FBS) to prevent the influence of hormones or estrogen-like compounds in the regular culture medium. The medium was changed every other day. The MCF-7 cells were then incubated with hormone free medium and treated by 0.001% DMSO (vehicle control group, C), 0.1 µM 17 β-estradiol (EM), 0.1, 1 or 10 µM of ferulic acid (equivalent to 0.0194, 0.194, and 1.94 mg/ml) ( FL, FM and FH), 0.0256, 0.256, and 2.56 mg/ml SWT (SL, SM and SH) for 6 hours. Three replicates for each of the eight treatment groups were included, resulting in a total of 24 samples (wells). The detailed experimental information including names and concentrations of the treatments are shown in **Figure 1** and **Table 1**.

### RNA extraction

Total RNA were extracted using RNeasy Mini Kit (QIAGEN, Valencia, California), following the manufacturer's protocol. The concentrations of RNA were measured by a NanoVue Plus (GE Healthcare, Piscataway, NJ, USA) and adjusted to 0.2 µg/µl. The RNA samples were stored at −80°C before further processing for microarray analysis.

### Microarray processing

RNA quality was checked using the RNA 6000 LabChip and Agilent 2100 BioAnalyzer. Only high quality RNA with RNA Integrity Number (RIN) >9.0 were used for microarray experiments. The 24 RNA sample IDs were randomly ordered and were blinded to the microarray core facility, the Clinical Transcriptional Genomics Core at Cedars-Sinai Medical Center, in order to minimize the impact of potential confounding factors such as sample processing time and order. The gene expression data were generated using 24 Affymetrix Human Genome U133 Plus 2.0 arrays (Santa Clara, CA, USA). Each U133 Plus 2.0 array consists of 54,675 probe sets detecting over 47,000 transcripts.

The cRNA synthesis and labeling was carried out following Affymetrix one-cycle sample preparation protocol. Briefly, biotinylated cRNAs were prepared according to the standard Affymetrix GeneChip protocol. One µg of total RNA from each sample, along with poly A spikes (labeling control), were converted to double-stranded cDNA with GeneChip One-Cycle cDNA synthesis kit (Affymetrix). After second-strand synthesis, the cDNA was purified with the GeneChip sample cleanup module (Affymetrix). Biotinylated cRNAs were synthesized by in vitro transcription using the Affymetrix GeneChip 3′-Amplification kit. The A260/280 ratio and yield of each of the cRNAs were obtained. For each sample, 10 µg of biotinylated cRNA along spiked controls (bioB, bioC, bioD and cre) was hybridized to a Human Genome U133 Plus 2.0 array for 16 hours at 45°C. Following hybridization, arrays were washed, stained and then scanned with an Affymetrix GeneChip® 3000 7G scanner.

### Real-time RT-PCR

To validate the microarray results, the MCF-7 cells were treated in a separate experiment using the identical experiment conditions and method as described above. One microgram of total RNA was incubated with DNase I, and reverse-transcribed with oligo dT using Superscript II RT-PCR (Invitrogen). One microliter of RT product was amplified by primer pairs specific for *HMOX1, NQO1, SLC7A11*, *GCLM* and *GCLC*. The *GAPDH* gene was used as a normalizing control. The primer sequences are available upon request. Relative gene expression was measured using the GeneAmp 7300 Sequence Detection system (Applied Biosystems, Foster City, CA, USA) using a SYBR Green protocol. For all amplifications, a standard amplification program was used (1 cycle of 50°C for 2 min, 1 cycle of 95°C for 10 min, 50 cycles of 95°C for 15 s and 60°C for 1 min). At the end of PCR cycling steps, data for each sample was displayed as a melting curve. The ABI SDS software was used to determine a “Threshold Cycle' (Ct), which was the cycle number where the linear phase for each sample reached the threshold detection level.

### Luciferase reporter gene assay

The luciferase reporter construct pGL4.22-ARE was a gift from Dr. Donna Zhang at the University of Arizona. It was generated by cloning a 39-bp antioxidant responsive element (ARE)-containing sequence from the promoter region of the NAD(P)H quinone oxidoreductase 1 (NQO1) into the pGL4.22 vector (Promega, Madison, WI, USA) [48]. The HEK293 or MCF-7 cells were transfected with the pGL4.22-ARE plasmid and a constitutively active renilla luciferase (pRL-TK-luc, from Promega; to correct for tranfection efficiency) (40∶1 ratio) using FuGENE HD Transfection Reagent (Roche Applied Science, Indianapolis, IN, USA) according to the manufacturer's instructions. Twenty-four hours after transfection, the cells were exposed to the extracts of SWT or four herbal components for another 24 hours. Cell lysates were used for determining luciferase activities of both firefly and renilla by the dual luciferase reporter gene assay (Promega). Firefly luciferase activity was normalized to renilla luciferase activity. The experiment was carried out in triplicate and expressed as the mean ± SD.

### Microarray data analysis

Microarray data specifically generated for this study are MIAME compliant. The raw data are available through the National Center for Biotechnology Information's Gene Expression Omnibus (GEO series accession number: GSE23610). The microarray gene expression data were imported to ArrayTrack [49], a software system developed by the U.S. Food and Drug Administration's National Center for Toxicological Research for the management, analysis, visualization and interpretation of microarray data (http://www.fda.gov/ArrayTrack/). The probe set-level expression data were summarized from probe-level data with Robust Multichip Average (RMA) [50] by taking all 24 microarrays together. Statistical testing and clustering analysis were conducted using ArrayTrack. Additional calculations were performed within JMP 7 (SAS Institute, Cary, NC, USA) and MATLAB 7.10 (MathWorks, Natick, MA, USA). For each probe set, log2-transformed intensity data were used in a two-sample t-test to obtain a *p* value and a fold change (FC).

### Quality assessment of the microarray data

Hierarchical clustering analysis combined with heatmap was applied to evaluate the overall reproducibility and variation of 3 replicates within each group and the differences among the 8 groups. The log_2_-transformed expression intensities of 54,675 probe sets with RMA summarized data from 24 microarrays were used to calculate the correlation coefficients between two gene expression profiles and construct the heatmap. The quality of microarray data generated in this study is excellent for identifying differentially expressed genes.

### Identification of differentially expressed genes

One important task in microarray data analysis is to identify a list of differentially expressed genes between two conditions (e.g. treatment versus control) [13], [14]. Many methods have been used for selecting differentially expressed genes and the choice of the “best” gene selection methods has been under extensive debate [15], [16]. In this study, genes differentially expressed between two sample groups were selected following the recommendations of the MAQC project [12], [51]. Briefly, a t-test *p* value and a fold change comparing a treatment group and the control were calculated for each probe set. Probe sets with a *p* value greater than a pre-defined cutoff (e.g. *p*>0.05) were removed and the remaining probe sets were ranked according to the magnitude of fold changes. Genes measured by the probe sets with a fold change (FC) greater than a pre-defined threshold (e.g. FC>1.5) were considered as differentially expressed. This straightforward approach of combining a non-stringent *p* value filtering with a fold-change based ranking has been found to generate more reproducible and reliable differentially expressed genes [12], [15], [16], [51], [52]. The expression profiles of the differentially expressed genes have been used as an input to search reference gene expression data (*i.e*., CMAP database) for treatments with similar expression profiles or to identify pathways enriched with the differentially expressed genes. To reduce the number of false-positive differentially expressed genes among 54,675 probe sets, we controlled for the false-discovery rate (FDR) at the level of 0.05 as described previously [53]. However, the FDR criteria of 0.05 may be too stringent as more than half of the genes identified using the above criteria would be removed. Thus, although the criteria of *p*<0.05 and FC>1.5 were used with low stringency, this is expected to detect more genes truly associated with SWT at the expense of increasing the number of false-positives to be validated by other bioinformatic and experimental means.

### Identification of pathways enriched with differentially expressed genes

The lists of differentially expressed genes were then imported to the Ingenuity Pathway Analysis (IPA) software (Ingenuity® Systems, www.ingenuity.com) to identify pathways that are enriched with the genes. The probe sets were mapped to the HUGO gene symbols within IPA software. When multiple probe sets map to the same HUGO gene symbol, only the probe set with the maximal absolute log2 fold change value was kept for identifying enriched IPA canonical pathways. Probe sets that do not map to any HUGO genes were discarded. For each canonical pathway, the Fisher's exact test *p* value was calculated to measure the statistical significance of enrichment of the pathway of the differentially expressed genes in relation with what would be expected by chance from the total number of unique genes in the input list. In addition, the ratio of the number of input genes that are in the pathway to the total number of genes in the pathway was calculated. The *p* value and the ratio indicate the levels of impact the treatment has on the pathway. The pathways were ranked according to *p* values, with the most significantly impacted pathways are shown on the top.

The lists of differentially expressed genes were also imported to the Kyoto Encyclopedia of Genes and Genomes (KEGG) database [54], [55], [56]. The probe sets in each treatment group were mapped to the HUGO gene symbols within the KEGG database, and the pathways enriched with the differentially expressed genes according to the Fisher's exact test *p* values are considered to be impacted by the treatments.

### Comparison of gene expression profiles with the CMAP reference database

To help better understand the underlying mechanisms of the therapeutic effects of SWT, we used the gene expression profiles of SWT treatments as queries to search the “Connectivity Map” (CMAP, http://www.broadinstitute.org/cMAP/) reference database (Build 02), which contains more than 7,000 expression profiles (instances) mainly from three cell lines (MCF-7, HL60 and PC3) treated with 1,309 compounds [17]. The query to CMAP is two lists of differentially expressed genes (listed as Affymetrix probe sets), one for up-regulation and the other for down-regulation, in which genes are ranked by the absolute log2 fold change values with descending order. The similarity between the gene expression profile of the query signature and that of a CMAP instance is measured by the connectivity score, ranging from -1 to 1. A high positive connectivity score indicates that the corresponding perturbagen in the CMAP database may similarly induce the expression change as the query agent. A high negative connectivity score indicates that the corresponding perturbagen in the CMAP database may reverse the expression effects of the query agent. Multiple profiles may exist for the same CMAP chemical name (due to different treatment concentrations, cell lines, or batches). CMAP allows individually matched instances consolidated by CMAP chemical name and cell line. Each combination of chemical names and cell lines combination comes with a mean connectivity score and a permutation *p* value, which indicates the probability of enrichment of a set of instances in a list of all instances by chance.
